# Psychometric properties of the 9-item Posttraumatic Cognitions Inventory (PTCI-9) in an Iranian sample

**DOI:** 10.47626/2237-6089-2022-0534

**Published:** 2024-11-06

**Authors:** Narges Barzgar, Hamid Poursharifi, Fereshte Momeni, Samaneh Hosseinzadeh

**Affiliations:** 1 University of Social Welfare and Rehabilitation Sciences Student Research Committee Tehran Iran Student Research Committee, University of Social Welfare and Rehabilitation Sciences (USWR), Tehran, Iran.; 2 USWR Department of Clinical Psychology Tehran Iran Department of Clinical Psychology, USWR, Tehran, Iran.; 3 USWR Biostatistics Department Tehran Iran Biostatistics Department, USWR, Tehran, Iran.

**Keywords:** Psychological trauma, cognition, inventories, psychometrics, PTSD

## Abstract

**Introduction:**

The Posttraumatic Cognitions Inventory (PTCI) is a widely used measure for assessing negative posttraumatic cognitions that are common among individuals with trauma-related disorders. There was a need for a valid and reliable short form of the PTCI in Persian.

**Objectives:**

This study aimed to translate the 9-item version of the PTCI (PTCI-9) into Persian and evaluate its characteristics and psychometric properties.

**Methods:**

This was a cross-sectional psychometric study using the translation and back-translation technique. Experts assessed the scale's content validity. Participants were 207 Iranian individuals recruited from the general population, 151 of whom were trauma-exposed. Participants completed the Persian version of the PTCI-9, the Beck Depression Inventory (BDI-II), and the World Health Organization Quality of Life (WHO-QOL) scale. The psychometric properties of the Persian version of PTCI-9 were assessed using exploratory and confirmatory factor analysis (CFA) methods. Cronbach's α coefficient and Pearson's correlation coefficients were also calculated.

**Results:**

Factor analyses supported a three-factor model including the Self, World, and Self-Blame subscales. The Cronbach's alpha of the Persian version of PTCI-9 (α = 0.74) and its subscales (0.76, 0.82, 0.78) demonstrated acceptable reliability. The Persian PTCI-9 also had strong test-retest reliability (r = 0.79). The correlations between the Persian version of the PTCI-9 and the BDI-II (r = 0.60) and the WHO-QOL (r = −0.54) indicated that the scale also has convergent validity.

**Conclusion:**

The Persian version of the PTCI-9 showed acceptable psychometric properties. It is a brief and pragmatic measure that can be used in Iranian trauma-exposed patients for research and clinical purposes.

## Introduction

Over 70% of people experience traumatic events worldwide.^1^ However, some of them (7-8%) develop posttraumatic stress disorder (PTSD). The underlying mechanism of PTSD is the posttraumatic cognitions that are the main factors contributing to developing and maintaining PTSD. One of the most widely used instruments for assessing these cognitions is the Posttraumatic Cognitions Inventory (PTCI).^2^ Although, a number of studies have investigated its psychometric properties, the new short-form of the PTCI with 9 items (PTCI-9) has not been fully studied yet. Moreover, it needs to be translated into the Persian language and the structure, validity, and reliability of the resulting Persian version of scale should be evaluated.

### Trauma

Trauma has been defined in several contexts; trauma, as a non-ordinary event, is outside the range of human experience. Based on the universal emotional response, a trauma is a stressor that would evoke significant symptoms of distress in almost everyone.^3^ Although it was believed that trauma is limited to a specific list of events, such as earthquakes, assaults, and other specific events, trauma can be defined as an event that creates a threat to life or physical integrity and produces an intense emotional reaction. Among all the above definitions, the Shattered assumption theme conceives of trauma as an event that disrupts fundamental beliefs about self and world. These fundamental beliefs that are thus negatively altered are called posttraumatic cognitions. Posttraumatic cognitions refer to the negative thoughts and beliefs that occur after a traumatic experience.^4^ Although most people experience trauma in their lives, only a few of them develop PTSD or persistent PTSD symptoms; the difference is in their underlying cognitions. Traumatized individuals with more negative cognitions about self and the world and those with negative appraisals about the trauma and its consequences can be considered as having persistent PTSD. So, psychotherapy should correct these cognitions, with the result that the patients recover from PTSD.^2^

### PTCI

The PTCI is the most widely used measure of posttraumatic cognitions. Foa et al.^2^ developed this scale in 1999. The PTCI is a self-report measure that comprises 33 items with a seven-point Likert response scale and evaluates three main domains: Negative Cognitions about Self, Negative Cognitions about World, and Self-Blame. It differentiates traumatized individuals from those without PTSD. The PTCI detects PTSD patients correctly and correlates highly with PTSD severity. The scale's three domains can predict PTSD severity, depression, and anxiety very well.^2^

The instrument is also used in PTSD intervention research because one of the hallmarks of successful treatment for PTSD is that the treatment can change posttraumatic cognitions. In one study, for example, after trauma-focused cognitive-behavioral therapy, trauma-related maladaptive appraisals (assessed using the PTCI) changed and consequently symptoms of posttraumatic stress disorder decreased. Both changes (change in cognitions and change in PTSD symptoms) were correlated; changes in the cognitions were anticipated and predicted a reduction in the symptoms of the disorder.^5^ The PTCI has also been utilized to compare the effectiveness of trauma-related therapies. A more recent study compared three short treatments: eye movement desensitization and reprocessing (EMDR); stress management focused on trauma; and psychological first aid. In this study, the PTCI was one of the three main tools used, besides the PTSD checklist and the Beck Depression Inventory (BDI-II).^6^ Since treatment of PTSD is associated with significant improvements in posttraumatic cognitions, changes in PTCI scores can predict the decline in PTSD symptoms.^7^

Various studies have examined the psychometric properties of the PTCI in a variety of populations, including adolescents,^8^ people who have had vehicle accidents,^9^ women who have experienced sexual violence,^10^ and those who have experienced interpersonal trauma. This questionnaire has been used widely and has been translated and reviewed in various languages including Turkish,^11^ German,^12^ Korean,^13^ and Chinese.^14^

### PTCI-9

There was a need for a more pragmatic, brief, and easy to administer measure because the original PTCI is not very practical for busy clinicians. Foa et al.^2^ suggested that the PTCI could be shortened to make it more useful for research and clinical applications. Therefore, in 2019, Wells et al.^15^ developed a short form of the PTCI. The shortened version consists of only nine items and can reduce patient and therapist burden.^15^ The items in the original item pool that Foa et al. compiled for PTCI represented nine different concepts. These nine concepts were: general negative view of self; perceived permanent change; alienation from self and others; hopelessness; negative interpretation of symptoms; self-trust; self-blame; trust in other people; and unsafe world.^2^ The nine items that comprise the PTCI-9 represent these main concepts. The PTCI-9 is more practical for use in research and clinical settings. It is also useful in electronic health records because it can reduce the burden on patients and health providers. Notably, a French version of the PTCI-9 has been developed and studied recently (α = 0.78 to 0.80).^16^

Although the PTCI-9 is short and user-friendly, it cannot be used with the Iranian population yet, because its psychometric features in the Iranian population were unclear until now. This study aimed to translate the PTCI-9 into the Persian language and report the resulting scale's psychometric properties in the Iranian community.

## Methods

### Participants and procedures

For this cross-sectional study in the Iranian population, a sample of 207 individuals were recruited by voluntary sampling. In the whole sample, there were 46 men (22%) and 161 women (78%). The average age was 32 (± 10) years.

First, the English version of the PTCI-9 was translated to Persian. The first draft of the translated scale was given to five non-psychology college students. They were asked to read the items and determine if all sentences were clear and meaningful. The back-translation process was conducted by an English language expert. Three psychology scholars compared the Persian and the English versions and confirmed the translation had been correctly conducted (after requesting a few minor revisions).

Since the study was conducted during a coronavirus quarantine period, data gathering was conducted online. From an overall sample of 398 visitors, 264 individuals participated and a sample of 207 Iranian participants fully answered the Persian version of the PTCI-9, the BDI-II, and the WHO-QoL questionnaire. The response rate was estimated at 78%. Additionally, 27 individuals participated in a 1-week retest.

### Measures

PTSD Checklist for the Diagnostic and Statistical Manual of Mental Disorders, 5th edition (DSM-5) (PCL-5)

The PTSD Checklist for the DSM-5 is a self-report measure including 20 items.^17^ It is widely used to assess PTSD symptoms based on DSM-5 criteria. Each item is rated on a Likert scale from 1 ("not at all") to 4 ("extremely"). The Persian version of the PCL-5 has acceptable psychometric properties.^18^ In this study, the symptom questions were excluded. So only questions 1 and 2 remained to assess traumatic experiences and their time of occurrence.

#### Posttraumatic cognitions (PTCI-9)

Wells et al.^15^ developed the short form of the PTCI consisting of nine items to briefly measure negative cognitions which are common after trauma. It assesses three domains of cognition including Cognitions about Self (three items), Cognitions about World (three items), and Self-Blame (three items). The items are rated on a Likert-type scale from 1 (Totally disagree) to 7 (Totally agree). Wells demonstrated high reliability and validity of the PTCI-9 in Veteran samples. The Cronbach's alpha for the total scale was 0.87, and the internal consistency of each of its subscales was also high – Self (α = 0.83), World (α = 0.85), and Self-Blame (α = 0.80). The PTCI-9 significantly correlated with the PTCI and with other measures including the Clinician-Administered PTSD Scale (CAPS-5) (r = 0.48, p < 0.01), the PTSD Checklist (r = 0.58, p < 0.01), the BDI-II (r = 0.67, p < 0.01), and the Quality of Life Inventory (QOLI) (r = −0.51, p < 0.01).^15^ In this study, the Persian version was used to evaluate factors of the Persian version of PTCI-9 and its psychometric features.

#### BDI-II

The BDI-II consists of 21 items.^19^ Each section assesses depression symptoms, which are scored from 0 to 3. The Persian version of the BDI-II is valid and reliable.^20^ In this study, the Persian version of the BDI-II was used to measure the presence and severity of depression in participants and to illustrate the correlation between posttraumatic cognitions and depression severity as evidence of convergent validity.

#### World Health Organization Quality of Life Brief (WHOQOL-BREF)

The WHOQOL-BREF form consists of 26 items.^21^ Two items assess perceived quality of life (QoL) and overall health satisfaction, and the other 24 items measure QoL in four domains of physical health, psychological well-being, social relationships, and environment. Higher scores on this scale demonstrate higher QoL.^22^

### Data analysis

To examine the latent structure of the scale, exploratory factor analysis (EFA) was conducted using IBM SPSS 23.0 and confirmatory factor analysis (CFA) was conducted with the R 3.5.1 Lavaan package. The sample was randomly divided into two subsamples, with 103 and 104 individuals in each group. Exploratory factor analysis was conducted using principal component analysis and the varimax rotation method, to not allow correlations between factors. The Kaiser Meyer Olkin test (KMO) and Bartlett's test were applied. The internal consistencies of subscales were assessed with Cronbach's alpha coefficients. The convergent validity of the scale was assessed with average variance extracted (AVE) and composite reliability (CR).

Confirmatory factor analysis was used to confirm the factors extracted with EFA. The model's fit was assessed with the root mean square error of approximation (RMSEA) and the standardized root mean square residual (SRMR), which should be smaller than 0.08, Bentler's comparative fit index (CFI), which should be larger than 0.90, and the Sattora-Bentler χ^2^(S-B χ^2^), which should not be statistically significant.

To investigate the test-retest reliability, 27 people completed the PTCI-9 twice, with a retest interval of 1 week. Moreover, the Pearson coefficient was calculated for the correlation with BDI-II scores to assess convergent validity. The study also calculated the correlation between the PTCI-9 and the WHO-QoL.

The Research Committee of the University of Social Welfare and Rehabilitation Sciences endorsed this research and the ethical code for this study is IR.USWR.REC.1399.174.

## Results

As Table 1 shows, 108 individuals were married and 99 were not. Most participants had a Bachelor of Arts (37%) or Master of Arts (32.4%) degree. Most participants were students (33.3%) or employers (21.3%). The participants recruited were residents of various areas of Iran, including Tehran (20.8%) and Mashhad (35.3%) mostly.

**Table 1:** The socio-demographic characteristics of Iranian participants who answered the Persian version of the 9-item Posttraumatic Cognitions Inventory (PTCI-9) (n = 207)

Characteristic		Trauma	No trauma	Full sample
Gender	
	Female	121 (80.1)	40 (71.4)	161 (78.0)
	Male	30 (19.9)	16 (28.6)	46 (22.0)
Marital status				
	Single	71 (47.0)	20 (35.7)	91 (44.0)
	Married	74 (49.0)	34 (60.7)	108 (52.2)
	Divorced/widowed	6 (4.0)	2 (3.6)	8 (3.9)
			
Highest educational level				
	No diploma	7 (4.6)	4 (7.1)	11 (5.3)
	Diploma	24 (15.9)	4 (7.1)	28 (13.5)
	Upper diploma	7 (4.6)	5 (8.9)	12 (5.8)
	Bachelor of Arts	57 (37.7)	20 (35.7)	77 (37.2)
	Master of Arts	46 (30.5)	21 (37.5)	67 (32.4)
	Ph.D. and above	10 (6.6)	2 (3.6)	12 (5.8)
			
Job-status				
	Housewife	20 (13.2)	8 (14.3)	28 (13.5)
	Student	52 (34.4)	17 (30.4)	69 (33.3)
	Employer	30 (19.9)	14 (25.0)	44 (21.3)
	Self-employed	20 (13.2)	9 (16.1)	29 (14.0)
	Other	23 (15.2)	5 (8.9)	28 (13.5)
	Not employed	6 (4.0)	3 (5.4)	9 (4.3)
			
Area of residence				
	Tehran	32 (21.2)	11 (19.6)	43 (20.8)
	Masshhad	51 (33.8)	22 (39.3)	73 (35.3)
	Isfahan	5 (3.3)	4 (7.1)	9 (4.3)
	Ahwaz	4 (2.6)	3 (5.4)	7 (3.4)
	North of Iran	6 (4.0)	1 (1.8)	7 (3.4)
	Other areas	53 (35.1)	15 (26.8)	68 (12.0)
			
Diagnosis*				
	None	134 (88.2)	48 (87.3)	182 (87.9)
	Schizophrenia spectrum	1 (0.7)	0 (0.0)	1 (0.5)
	Bipolar disorder	1 (0.7)	0 (0.0)	1 (0.5)
	Major depressive disorder	6 (4.0)	1 (1.8)	7 (3.4)
	Anxiety disorder	7 (4.7)	5 (9.1)	12 (5.8)
	OCD	2 (1.3)	1 (1.8)	3 (1.4)
	PTSD	1 (0.7)	0 (0.0)	1 (0.5)

Data presented as n (%). Mean age of participants was 32 years (standard deviation [SD] = 10 years).

* Reflects the participants’ answer to the self-report question "Do you suffer from a mental illness?"

Seventy-one participants (34.3%) had experienced relational traumas. Others reported loss of loved ones (10.1%), academic/job-related trauma (8.7%), difficult illness (4.8%), childhood trauma (4.3%), sexual assaults (2.9%), and accidents (2.9%) respectively. Also, 10 participants (4.8%) reported other traumatic experiences including dog attack, war, physical fight, and insults from a father or a family member. Twelve participants did not respond to the trauma question for personal reasons, and the traumatic experiences of 44 participants were only normal hassles. Moreover, 56 (27%) participants reported no traumatic experiences. Analysis of variance (ANOVA) and the least significant difference (LSD) post-hoc test revealed that participants with "relational trauma" differed from difficult illness, loss, and childhood trauma in terms of posttraumatic cognitions.

Descriptive statistics were calculated to derive the mean and standard deviation (SD) for posttraumatic cognitions, depression, and QoL. The average score for posttraumatic cognitions in the Iranian sample was 3.35 (SD ± 1.07), with higher scores on the World subscale (4.45 ± 1.51). The average depression score on the BDI-II was 14.87 (SD = ± 11.77). The mean QoL score was 74.14 (mean scores for the subscales physical health, psychological wellbeing, social relationships, and environment were 19.79, 18.91, 9.52, and 26.00 respectively).

**Figure 1:**
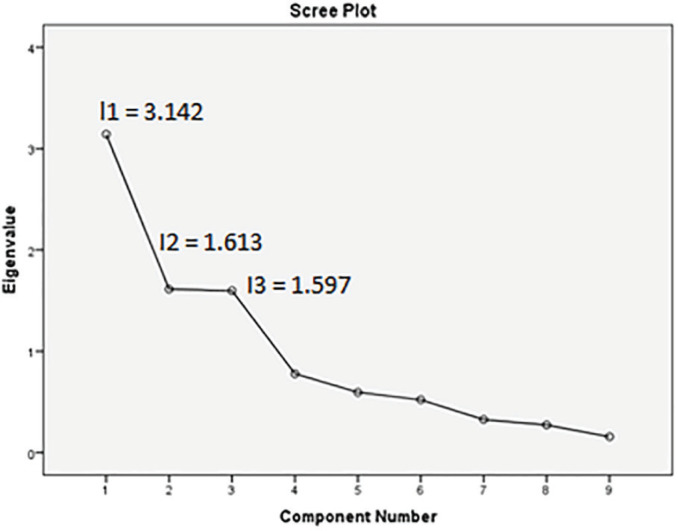
The scree plot indicating three factors with eigenvalues larger than 1 for the Persian version of the 9-item Posttraumatic Cognitions Inventory (PTCI-9), extracted by exploratory factor analysis (EFA) from data for a random subsample of Iranian people (n = 103). The rotated solution eigenvalues for the three components are 2.384, 2.285, and 1.682. The three factors explained 34.909, 52.828, and 70.571 cumulative percentage of variance for loading 1, 2, and 3, respectively.

For the EFA, the responses of a random subsample of 103 individuals were used to conduct the EFA for the scale. The KMO test indicated very good sampling adequacy (0.654). Bartlett's test was significant (chi-square value = 354.206, p < 0.05), showing that the items are correlated and factor analysis is appropriate. Latent factors were extracted by principal components analysis. The factors extracted were rotated with the varimax method. This criterion suggested a three-factor structure for the PTCI-9, which explained 70.6% of the variance (Figure 1).

The matrix pattern of the extracted factors is shown in Table 2. The first subscale (l1) is Cognitions about Self, with items 3, 5, 8, and 9, the second subscale (l2) is Cognitions about World with items 2, 4, and 6, and the last (l3) is Self-Blame, with items 1 and 7 (Table 3).

**Table 2:** The internal consistency of the Persian version of the 9-item Posttraumatic Cognitions Inventory (PTCI-9) in an Iranian sample

Components	Cronbach's alpha	AVE	CR
l1: Cognitions about Self	0.761	0.730	0.746
l2: Cognitions about World	0.827	0.864	0.870
l3: Self-Blame	0.788	0.898	0.901

AVE = average variance extracted; CR = composite reliability; l1 = loading 1; l2 = loading 2; l3 = loading 3.

**Table 3:** Factor analysis of the Persian version of the 9-item Posttraumatic Cognitions Inventory (PTCI-9) in the Iranian subsample (n = 103)

PTCI-9 item		Factor loading		
		1	2	3
Factor 1: Cognitions about Self				
	3. Somebody else would not have gotten into this situation.	**0.670**	-0.017	-0.127
	5. I have no future.	**0.747**	0.252	0.122
	8. I feel like I don't know myself.	**0.685**	0.175	0.172
	9. Nothing good can happen to me anymore.	**0.910**	0.080	0.033
			
Factor 2: Cognitions about World				
	2. People can't be trusted.	0.098	**0.902**	0.052
	4. I can't rely on other people.	0.235	**0.881**	-0.054
	6. People are not what they seem.	0.061	**0.757**	0.111
			
Factor 3: Self-Blame				
	1. The event happened because of the way I acted.	-0.003	-0.038	**0.910**
	7. There is something about me that made the event happen.	0.107	-0.141	**0.880**

The extraction method was principal component analysis with varimax rotation. Factor loadings above 0.50 are shown in bold. Adapted from Wells et al.^15^

The internal consistency of these three factors was assessed with Cronbach's alpha coefficients, all of which were larger than 0.7, showing good consistency (Table 2). The internal consistency for the whole scale is 0.739. Additionally, AVE > 0.5, CR > 0.7, and CR > AVE for all three subscales, showing that the convergent validity of the scale is appropriate (Table 2).

**Figure 2:**
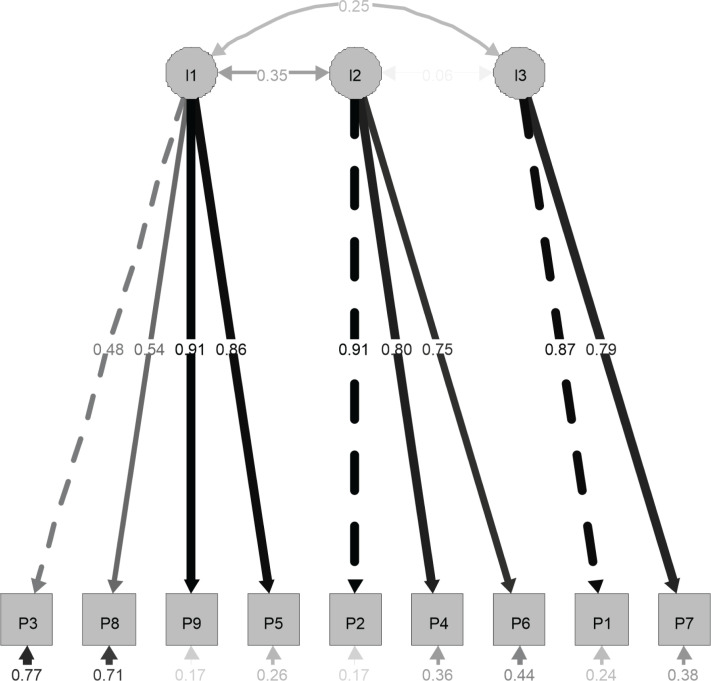
Factor loadings for the Persian version of the 9-item Posttraumatic Cognitions Inventory (PTCI-9) in the 104 subject Iranian subsample. n = 104; I1 = loading 1, P1 = item 1.

Confirmatory factor analysis was conducted to confirm the latent structure of the scale (Figure 2). This analysis was carried out on the second subsample (n = 104). The values related to the goodness of fit indices of the model are in the optimal range. The chi-square statistic value was 41.912 (degrees of freedom [df] = 24), the robust CFI is equal to 0.954, the Tucker Lewis index (TLI) is 0.931, the SRMR is 0.076, and the robust RMSEA is 0.085 (0.039, 0.127; 90%Cl). This model has the best indices, showing that the three-factor model extracted is the best representation of the structure of the data.

The test-retest correlation of PTCI-9 scores showed acceptable test-retest reliability. Pearson correlations revealed high correlations for the whole scale and all three factors: R (total score) = 0.79, R (Self subscale) = 0.80, R (World subscale) = 0.79, R (Self-Blame subscale) = 0.68. They showed acceptable test-retest reliability over an interval of 7 days.

The Pearson correlation between PTCI-9 and BDI-II scores was significant (r = + 0.60, p = 0.05). The correlation between PTCI-9 and WHO-QOL was negative and significant (r = −0.50, p = 0.05). The PTCI-9 was significantly correlated with depression and QoL and therefore showed convergent validity in the current study.

## Discussion

This study aimed to evaluate the psychometric features of the Persian version of the PTCI-9 in the Iranian population. We studied the PTCI-9 because not only has the PTCI-9 been found to have better structural validity than the PTCI with adequate psychometric properties,^23^ but it is also a shorter measure. The data supported the three-factor structure model fit to the three subscales Negative Cognitions about Self, Negative Cognitions about World, and Self-Blame. We reported the mean and SD for the total score and the three subscales. The reliability and validity of PTCI-9 were also assessed in the Iranian general population during a period of coronavirus disease 2019 (COVID-19) pandemic lock down.

### Structure

The analysis revealed that the three-factor structure had the best fit to the data. This is the same structure as the model reported for the PTCI-9 by Wells et al.^15^ and by previous studies that have confirmed three-factor structures.^15,16,23,24^ Although the literature supports the three-factor model for the PTCI-9, the PTCI did not achieve a consistent factor structure or consistent validity in recent studies.^23^ Before development of the PTCI-9, the PTCI had been used with certain revisions. For example, researchers in Northern Ireland removed eight items (resulting in a PTCI with 25 items), researchers in Korea removed five items (resulting in a PTCI with 28 items), and researchers in China removed four items (resulting in a PTCI with 29 items) to achieve structures with adequate fit.^8,13,14^ Currently, there is agreement that the PTCI-9 is a promising alternative measure to the PTCI.^23,24^ However, whereas in the Wells et al.^15^ study, all three subscales of the original PTCI-9 had three items; in the present study, item 3 loaded onto the Self subscale instead of the Self-Blame subscale. As a result, the Self subscale contained four items and the Self-Blame subscale only had two items. This three-factor solution fitted the data without excluding any items and had the best indices for CFI, TLI, RMSEA, and SRMR. For the English version of the PTCI-9, Wells et al.^15^ found an acceptable fit, meeting the recommended CFI and SRMR cutoffs and coming very close to meeting the cutoff for RMSEA. The different result for the third item ("Somebody else would not have gotten into this situation") might be due to the difference in languages, since this sentence does not seem to convey a concept of self-blame in the Persian language. As the PTCI and PTCI-9 are culture-based measures, the structural model and the items involved are highly affected by culture and language. In fact, it seems that the low factor loading of item 3 onto factor 1 was due to the cultural and linguistic incompatibility of this item with Iranian society. Indeed, the factor structure model of the PTCI-9 needs some revisions in different countries and cultures, as was the case for the PTCI. In general, the PTCI-9 is highly context-related.

### Reliability

The Persian version of the PTCI-9 demonstrated strong internal consistency (α = 0.74); in common with the French version of the PTCI-9 that was developed and studied recently (α = 0.78 to 0.80).^16^ Internal consistency was confirmed with Cronbach's alpha for the whole scale and all three subscales. This finding supported the original study by Wells et al.^15^ who found strong internal consistency (α = 0.87) and also strong associations between PTCI-9 scores and PTSD measures. Consequently, they concluded that the PTCI-9 is a part of the PTSD diagnosis. Test-retest reliability was acceptable for the total scale, the World subscale, and the Self subscale and test-retest reliability was adequate for the Self-Blame subscale. The Wells et al.^15^ study did not report its test-retest reliability, but the original PTCI with 33 items had shown great test-retest reliability over a 1-week interval.^2^ The current study duplicated that and observed the same results. Based on psychometric studies of the PTCI (not the PTCI-9) in other languages, the Dutch version has shown sufficient validity and reliability (α = 0.95); as did the Korean version (α = 0.97) and the Turkish version (α = 0.93).^11–13,25^ It is necessary for the psychometric features of the short version (PTCI-9) to be studied in other countries, based on their context of culture and language. Moreover, the analysis showed that those participants with "relational trauma" differed from those with difficult illness, loss, and childhood traumas in terms of posttraumatic cognitions. In fact, the average PTCI-9 score was significantly higher among participants with relational trauma. Further studies with various distinct trauma-exposed samples are required to identify the reliability status of posttraumatic cognitions in different types of traumas.

### Validity

Face validity and content validity of the Persian version of the PTCI-9 were acceptable since five non-psychology students filled out the first draft and were able to fully understand it. Afterwards, three professors of psychology commented on the translation and back-translation. After some minor corrections were conducted, these experts reached a consensus. No items from the English version of the PTCI-9 were excluded. Previous studies have used comparison with PTSD and depression measures to verify the convergent validity of the PTCI. The present study showed the convergent validity of the Persian PTCI-9 was impeccable, according to the significant correlation between the PTCI-9 and depression scores. In addition to studies by Foa et al.^2^ and Wells et al.,^15^ other studies have also shown significant correlations between the PTCI and depression. Sexton,^26^ for example, demonstrated significant correlations between the PTCI and measures of PTSD and depressive symptoms. Although they used the original PTCI with 33 items, they found four factors in a sample of traumatized military veterans. They confirmed its convergent validity through good-to-excellent correlations with PTSD, depression, and resilience.^26^ In a Korean study of the PTCI with female victims of sexual violence, Shin et al. showed the PTCI was correlated with depression and anxiety symptoms.^13^ The association between the PTCI-9 and depression measure indicates that the PTCI-9 may be useful in examining how posttraumatic cognitions relate to depressive symptoms after exposure to a traumatic event. The three factors identified (Negative Cognitions about Self, Negative Cognitions about World, and Self-Blame) suggest a similarity with the cognitive triad of depression from Beck's paradigm (view of self, world, and future). For example, Shin et al.^13^ found that self-blame seemed to be more related to depression and anxiety rather than trauma symptoms. Moreover, depressive symptoms were more significantly correlated with "negative cognitions about the self" than with "negative cognitions about the world," while certain PTCI-9 items overlap with general depressive thinking (e.g., "Nothing good can happen to me anymore"). These results may suggest that the PTCI-9 can assess cognitions of trauma-related depression. Therefore, the PTCI-9 is a standardized brief version of the measure that can be used in PTSD and trauma-related depression research. Convergent validity was also demonstrated in this by the significant correlation between the PTCI-9 and the WHO-QOL.

Individuals who have experienced trauma are prone to develop symptoms of posttraumatic stress disorder.^27^ In such situations, the PTCI-9 can be used as an appropriate screening tool to help identify people prone to developing PTSD. Using such tools leads to more accurate evaluation, follow-up, and even prevention in people with traumatic experiences. The Persian version of the PTCI-9 with its great psychometric features may be useful in clinical and research settings, especially in the age of the COVID-19 pandemic. In a nutshell, the Persian version of the PTCI-9 is a standardized brief version of the measure that can be used in PTSD and trauma-related research with Persian-speaking populations.

### Limitations and suggestions

In this community-based study, the participants were not divided based on PTSD or trauma, so further psychometric studies are recommended investigating PTSD in different types of traumatized populations. The instrument could be evaluated in a study with an appropriate sample size in different clinical and non-clinical groups. Further validation of the PTCI-9 needs to be conducted by administering this shortened measure to participants to ensure that the factors identified here have practical and clinical significance. Given previous findings that posttraumatic cognitions vary according to the type of traumatic event an individual has experienced,^2^ it is necessary to evaluate the scale's factor structure, validity, and reliability in specific samples (e.g., individuals who have experienced a relational trauma) to ascertain if the PTCI-9 performs similarly across all samples and can therefore be utilized across all types of samples.

In the Persian version of the PTCI-9, item 3 did not load onto the self-blame factor. Hence, to overcome this limitation, there is a need to adapt item 3 ("Somebody else would not have gotten into this situation") so that Persian-speaking populations will easily perceive it as reflecting a self-blame concept. Alternatively, another item that does represent self-blame to a Persian speaking population could be developed to replace item 3. Further validation of the PTCI-9 needs to be conducted by administering this shortened measure to participants to ensure that the factors identified have practical and clinical significance. Further replication of the PTCI-9's psychometric properties is suggested.
